# Zoledronic Acid Regulates Autophagy and Induces Apoptosis in Colon Cancer Cell Line CT26

**DOI:** 10.1155/2017/7203584

**Published:** 2017-12-31

**Authors:** Jinhua Zhu, Meihui Liu, Yuanfen Liu, Yiting Zhang, Bing Yang, Wei Zhang

**Affiliations:** ^1^Jiangsu Health Vocational College, Nanjing, Jiangsu, China; ^2^College of Medicine, Nanjing University, Nanjing, Jiangsu, China; ^3^School of Pharmacy, Nanjing University of Chinese Medicine, Nanjing, Jiangsu, China

## Abstract

Zoledronic acid (ZOL) is the third generation of bisphosphonates, which can inhibit many tumors growth, especially to inhibit the growth of colon cancer. However, the molecular mechanism is still very mysterious. In this study, we observed that ZOL could regulate CT26 colon cancer cells autophagy, promote CT26 cells apoptosis, and inhibit CT26 cells proliferation. Western blotting analysis showed that proapoptosis protein caspase-3 was basically unchanged, whereas the expression of the activated caspase-3 was significantly increased, after CT26 cells were treated with different doses of zoledronic acid. Western blot also showed that ZOL could significantly affect the expression of p-p53 and autophagy-related proteins beclin-1 and p62. In conclusion, the antitumor effect of ZOL on CT26 colon cancer cells in vitro is achieved by apoptosis induction and autophagy regulation, resulting in inhibition of cell proliferation.

## 1. Introduction

Colorectum is one of the 5 most common sites of cancer diagnosed worldwide. Colon cancer threatens people's health and life severely with morbidity up to 5.5% in males and 7.7% females in China in 2012. As the current therapeutic regime seems unsatisfactory, exploring new therapeutic modalities is necessary.

Zoledronic acid (ZOL) is a third generation of bisphosphonate (BP) molecular class and an important weapon against osteoporosis and bones diseases [1–4]. It is reported that zoledronic acid has antitumoral activity on many human cancers with the help of growth factor release, cell adhesion, apoptosis, and autophagy [5–9]. Studies have investigated that the occurrence and development of colon cancer are not only related to the malignant transformation and overproliferation of cells, but also related to the reduction of apoptosis and autophagy [10–12]. However, the molecular mechanism of ZOL inhibiting colon cancer cell growth is still not clear.

In this study, colon cancer cell line CT26 was selected to investigate the effects of ZOL on its proliferation in vitro. We found that ZOL inhibited CT26 cells growth by inducing apoptosis and regulating autophagy.

## 2. Materials and Methods

### 2.1. Cell Culture

Colon cancer cell line CT26 was originally obtained from American Type Culture Collection (ATCC, Manassas, VA, USA) and was preserved and cultured by this laboratory. Cells were cultured in DMEM medium (Hyclone), supplemented with 10% fetal bovine serum (Hyclone) in 5% CO_2_ at 37°C.

### 2.2. Cell Viability Assay

Cells growing at the exponential (logarithmic) phase were digested with 0.25% trypsin and single cell-suspension solution was prepared. A total of 8 × 10^3^ cells per well were seeded in 96-well plates for 24 h and then treated with various concentrations of zoledronic acid (sigma, USA) for 24 h. The cell viability was measured using a commercial CCK-8 kit (Dojindo, Kumamoto, Japan) via colorimetric method according to the manufacturer's instructions.

### 2.3. Cell Apoptosis Analysis

Annexin-V FITC and PI double staining kit (Invitrogen, Grand Island, NY, USA) was used to analyze ZOL-induced cell apoptosis. After being cultured with medium containing ZOL at 0 *μ*M, 1 *μ*M, 10 *μ*M, 100 *μ*M, 200 *μ*M, and 300 *μ*M for 24 h, CT26 cells were collected and resuspended with 1 mL 1x binding buffer solution. After being fixed with 4% paraformaldehyde, 5 *μ*L of annexin-V FITC and 500 *μ*L of PI were added to each tube, mixed well, and incubated in dark for 15 min. The cells were finally resuspended with 400 *μ*L of 1x binding buffer. The apoptotic cells were detected with flow cytometry (BD Biosciences, San Jose, CA, USA).

### 2.4. Western Blotting Analysis

CT26 cells were processed, respectively, for 12 h, 24 h, 36 h, and 48 h with a final concentration of 200 *μ*M ZOL. Then, separate CT26 cells and extract the total cytosolic proteins. Twenty micrograms (20 *μ*g) of proteins was dissolved by SDS-PAGE with 12% polyacrylamide-SDS gels followed by transfer onto PVDF membranes. After being blocked with 5% defatted milk powder solution for 1 h, the membrane was incubated overnight with Tris-buffered saline (TBS) containing anti-caspase-3, anti-c-caspase-3, anti-p-p53, anti-p62, anti-beclin-1 antibody or with anti-GAPDH antibody at 1 : 1000 dilution (Santa Cruz Biotech, Santa Cruz, CA, USA), respectively, and then incubated with goat-anti mouse IgG-horseradish peroxidase at 1 : 1000 as secondary antibody (Beyotime, Nantong, China). The membrane was incubated with ECL solution and was developed in X-ay film. The antibody band intensities of anti-caspase-3, anti-c-caspase-3, anti-p-p53, anti-p62, and anti-beclin-1 as well as anti-GAPDH following various treatments were scanned using gray scale scanning software.

### 2.5. Statistical Analysis

The experimental data were statistically conducted with SAS9.0 software. The change in apoptosis was analyzed with analysis of variance.

## 3. Results 

### 3.1. The Inhibitory Effects of Zoledronic Acid on the Proliferation of Colon Cancer Cells

**Figure 1:**
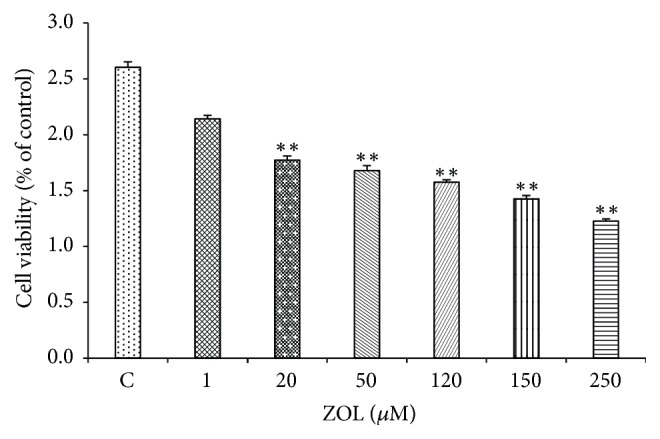
CT26 cells were exposed to the increasing ZOL concentrations (0–250 *μ*M) for 24 h and were then processed for MTS assay (*n* = 5). The bars represent the means ± SD from two independent experiments. ^*∗∗*^*p* < 0.01 versus the untreated control cells.

The inhibitory effects of zoledronic acid on the proliferation of colon cancer cells were detected by using CCK-8 kit. The results were shown in Figure 1. Zoledronic acid at varying concentrations all caused inhibitory effects on the proliferation of CT26 cells and the inhibitory effects became more noticeable with the increase in ZOL dose, displaying the clear dose dependence. The result indicated that zoledronic acid possessed obvious anticancer effects in vitro and displayed a significant dose-dependent relationship. With the increases in the doses of zoledronic acid, its inhibitory effects became more significant.

### 3.2. Effects of Zoledronic Acid on Apoptosis of Colon Cancer Cells

**Figure 2:**
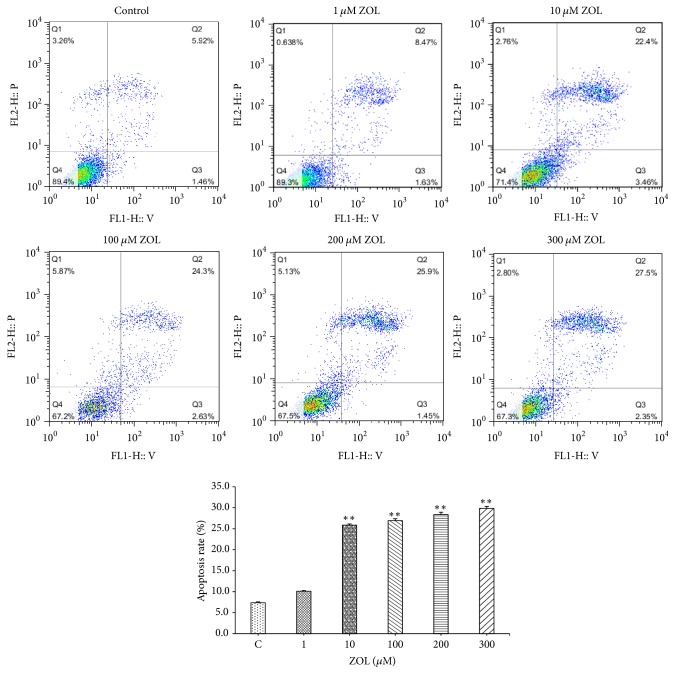
CT26 cells were exposed to the increasing ZOL concentrations for 24 h and were then processed for FCM (*n* = 5). The bars represent the means ± SD from two independent experiments. ^*∗∗*^*p* < 0.01 versus the untreated control cells.

Annexin-V method was applied for further investigation of ZOL-induced apoptosis. The results were shown in Figure 2; treatment of CT26 cells with zoledronic acid for 24 h induced apoptosis in a dose-dependent manner. With the increase in the doses of zoledronic acid, the proportions of the cells that undergone apoptosis were significantly increased.

### 3.3. Differential Expression of Apoptosis-Related Proteins in Colon Cancer Cells Induced by Zoledronic Acid

**Figure 3:**
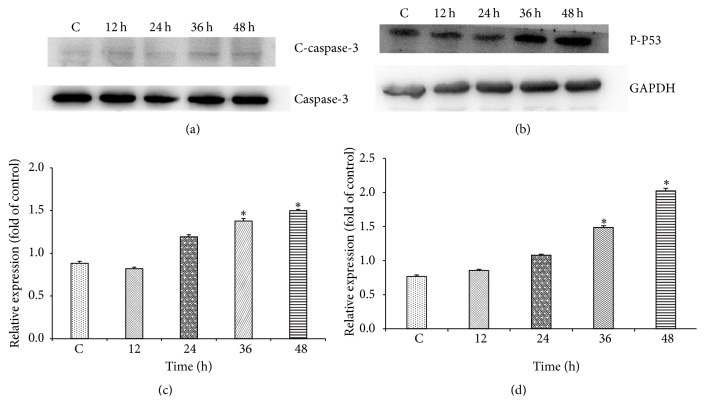
The cells were exposed to the 200 *μ*MZOL and at 12 h, 24 h, 36 h, and 48 h of exposure, the cell fractions were prepared and analyzed by 15% SDS-PAGE followed by Western blotting. (a and c) The level of c-caspase-3 in CT26 cells after being exposed to ZOL. (b and d) The level of p-p53 in CT26 cells after being exposed to ZOL. The data shown in C and D are the mean ± SD of the results of three independent experiments, respectively (^*∗*^*p* < 0.05 represents significant differences between the experimental and untreated control values).

Detection with Western blot revealed that zoledronic acid caused significant effects on the expression of apoptosis-related proteins. As shown in Figure 3, after being treated with 200 *μ*M ZOL for 36 h and 48 h, the relative expression of c-caspase-3 and p-p53 was significantly increased, indicating that ZOL-induced apoptosis is mediated via controlling the activation degree of caspase-3 and p-p53. It was positively related to time.

### 3.4. Differential Expression of Autophagy-Related Proteins in Colon Cancer Cells Induced by Zoledronic Acid

**Figure 4:**
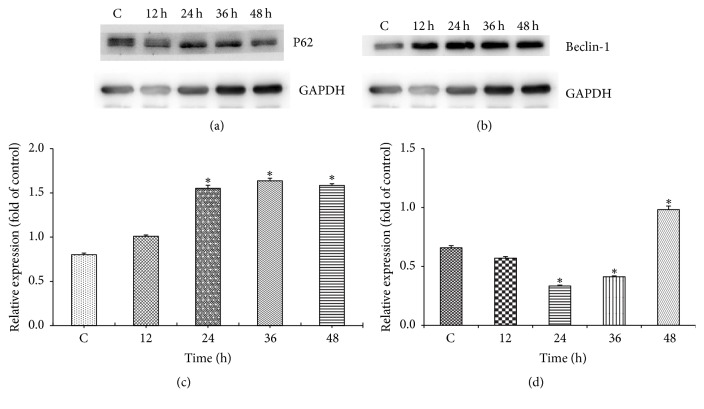
The cells were exposed to the 200 *μ*MZOL and at 12 h, 24 h, 36 h, and 48 h of exposure, the cell fractions were prepared and analyzed by 15% SDS-PAGE followed by Western blotting. (a and c) The level of P62 in CT26 cells after being exposed to ZOL. (b and d) The level of beclin-1 in CT26 cells after being exposed to ZOL. The data shown in C and D are the mean ± SD of the results of three independent experiments, respectively (^*∗*^*p* < 0.05 represents significant differences between the experimental and untreated control values).

Detection with Western blot revealed that zoledronic acid caused significant effects on the expression of autophagy-related proteins. As shown in Figure 4, after being treated with 200 *μ*M ZOL for 24 h, 36 h, and 48 h, the relative expression of p62 was significantly increased, whereas the expression of beclin-1 was obviously decreased and then increased significantly, suggesting that ZOL regulates autophagy.

## 4. Discussion

Zoledronic acid possesses a number of pharmacological functions including prevention of osteolytic lesions caused by tumors, reducing hypercalcemia induced by tumor and direct antitumor effect both in vitro and in vivo [13, 14]. However, its detailed anticancer mechanisms are still not clear. The results of this study indicate that ZOL at appropriate doses can directly inhibit the growth and proliferation, regulate autophagy, and induce apoptosis of colon cancer cells.

The tumorigenesis and metastasis are associated with the overproliferation of cells and the decrease of apoptosis, and tumor cells proliferating without limitation is the main feature. Cell apoptosis is an initiative cell death under gene regulation. With the deepening study of the tumor molecular biology, people can basically understand that the tumorigenesis and metastasis are also related to apoptotic dysfunction. Therefore, inducing tumor cells apoptosis has become a new direction of the treatment. So far, apoptosis signal pathways, which have been identified, include Fas/FasL pathway, caspase family pathway, cytochrome C signal pathway, and mitochondria pathway. Caspase family pathway is a very important apoptotic signaling pathway. Once the caspase family proteins are activated, irreversible protein degradation is initiated resulting in the degradation of the substrate protein upon induction of apoptosis. Caspase-3 is one of the most important members of the apoptotic family, because it is at the core of cell apoptosis and can react with many different substrates. Caspase-3 is also a common downstream regulator of the effector pathway of apoptosis, which is the only way of caspase cascade. Caspase-3 exists in many tissues and cells as an inactive zymogen, which will be cut into the active caspase and play a role in apoptosis after being stimulated by different apoptotic signaling [15]. P53 gene plays an important role in preventing cell proliferation and maintaining the integrity of DNA genome which was damaged. With the expression increasing, it will cause cell apoptosis directly or cause apoptosis by regulating other apoptotic genes indirectly [16–18]. Therefore, p53 is also one of the key molecules in cell apoptosis pathway. Researchers have found that caspase-3 and p53 play a role in the occurrence, development, and metastasis of colorectal cancer [19].

The activity of caspase-3 during apoptosis was detected by Western blot. Experiment proves that cleaved caspase-3 was increased during apoptosis. It suggests that caspase-3 is involved in the regulation of apoptosis in colon cancer cells. It is visible that the increase of cleaved caspase-3 expression plays an important role in the process of ZOL inducing colon cancer apoptosis. We found that the increased level of active caspase-3 protein, induced by ZOL, was time-dependent. The change of cleaved caspase-3 protein level promoted the apoptosis of colon cancer cells in vitro. In addition, activated caspase-3 works as a downstream protein mainly during late apoptosis. We have found that the apoptosis rate of late CT26 colon cancer cell was positively associated with the ZOL concentration. Thus, speculate that the increase of caspase-3 expression is also drug-dependent. At the same time, through detecting relative expression of p53, we found that the expression increased significantly by the time of exposure to ZOL. It shows that p53 was also involved in the regulation of cell apoptosis in colon cancer. ZOL, therefore, is likely to cause apoptosis of colon cancer cell through activating p53/caspase-3 signaling pathways. However, there are also a series of the intermediate links, which need further verification from animal.

Autophagy plays an important role in the occurrence, development, and metastasis of tumor. In the early stage of cancer, autophagy can maintain the homeostasis of the cell by removing the damaged mitochondria, peroxisomes, and other cytotoxic substances in normal cells, inhibiting the activation of oncogenes and preventing the occurrence of tumor. In advanced tumors, autophagy is an important reason for the drug resistance of tumor cells, leading to tumor cell survival and tumor cell growth promoting role in adverse circumstances, and can promote the proliferation and invasion and metastasis of malignant tumor. It has been reported that the treatment of colon cancer cells by knockdown of autophagy-related genes or the use of autophagy inhibitors will induce apoptosis through activation of p53 gene and endoplasmic reticulum stress in order to achieve antitumor effect [20]. Therefore, inhibition of autophagy may be an effective strategy for cancer therapy. Our experimental data shows that ZOL can significantly regulate the expression of autophagy-related proteins, and the expression of p62 was obviously upregulated, while the expression of beclin-1 was significantly changed. Our study has revealed that ZOL can regulate autophagy of colon cancer cells while inhibiting cell proliferation, suggesting that ZOL induces the abnormal autophagy and causes the promotion of tumor cell apoptosis and leads to antitumor biological function, which needed further study.

These results demonstrate that ZOL is an effective inhibitor of CT26 colon cancer cells. Its anticancer activities are mediated by induction of apoptosis and regulation of autophagy. In this study, we only conducted the experiments in vitro, but more in vivo experiments with animals needed to be conducted. Further observation on the blood concentration of ZOL within the patient's body are also needed to be done to investigate whether or not ZOL at this blood concentration can cause the same effects on colon cancer tissue and the related toxic side effects.
